# Astragaloside IV Protects against Isoproterenol-Induced Cardiac Hypertrophy by Regulating NF-κB/PGC-1α Signaling Mediated Energy Biosynthesis

**DOI:** 10.1371/journal.pone.0118759

**Published:** 2015-03-04

**Authors:** Suping Zhang, Futian Tang, Yuhong Yang, Meili Lu, Aina Luan, Jing Zhang, Juan Yang, Hongxin Wang

**Affiliations:** Key Laboratory of Cardiovascular and Cerebrovascular Drug Research of Liaoning Province, Drug Research Institute, Liaoning Medical University, Jinzhou, Liaoning, P.R. China; Temple University, UNITED STATES

## Abstract

We previously reported that Astragaloside IV (ASIV), a major active constituent of Astragalus membranaceus (Fisch) Bge protects against cardiac hypertrophy in rats induced by isoproterenol (Iso), however the mechanism underlying the protection remains unknown. Dysfunction of cardiac energy biosynthesis contributes to the hypertrophy and Nuclear Factor κB (NF-κB)/Peroxisome Proliferator-Activated Receptor-γ Coactivator 1α (PGC-1α) signaling gets involved in the dysfunction. The present study was designed to investigate the mechanism by which ASIV improves the cardiac hypertrophy with focuses on the NF-κB/PGC-1α signaling mediated energy biosynthesis. Sprague-Dawley (SD) rats or Neonatal Rat Ventricular Myocytes (NRVMs) were treated with Iso alone or in combination with ASIV. The results showed that combination with ASIV significantly attenuated the pathological changes, reduced the ratios of heart weight/body weight and Left ventricular weight/body weight, improved the cardiac hemodynamics, down-regulated mRNA expression of Atrial Natriuretic Peptide (ANP) and Brain Natriuretic Peptide (BNP), increased the ratio of ATP/AMP, and decreased the content of Free Fat Acid (FFA) in heart tissue of rats compared with Iso alone. In addition, pretreatment with ASIV significantly decreased the surface area and protein content, down-regulated mRNA expression of ANP and BNP, increased the ratio of ATP/AMP, and decreased the content of FFA in NRVMs compared with Iso alone. Furthermore, ASIV increased the protein expression of ATP5D, subunit of ATP synthase and PGC-1α, inhibited translocation of p65, subunit of NF-κB into nuclear fraction in both rats and NRVMs compared with Iso alone. Parthenolide (Par), the specific inhibitor of p65, exerted similar effects as ASIV in NRVMs. Knockdown of p65 with siRNA decreased the surface areas and increased PGC-1α expression of NRVMs compared with Iso alone. The results suggested that ASIV protects against Iso-induced cardiac hypertrophy through regulating NF-κB/PGC-1α signaling mediated energy biosynthesis.

## Introduction

Cardiac hypertrophy is a key compensatory mechanism in response to pressure or volume overload, involving some alterations in myocardial metabolism[1], inflammation[2], remodeling and neurohormonal activation. Sustained adrenergic receptor activation by Isoproterenol (Iso) is associated with cardiac hypertrophy[3]. A complex set of metabolic process is shown to be closely involved in the development of cardiac hypertrophy[4,5], subsequently resulting in the alterations in mitochondrial function and fuel metabolic abnormalities in the hypertrophied heart. Energy supply in the form of ATP is mandatory to sustain cardiac contractile and relaxation functions[6,7]. Both cardiac systolic and diastolic functions are dependent on mitochondrial-generated ATP, suggesting that mitochondrial bioenergetic decline contributes to the progression of hypertrophy[8]. ATP5D, subunit of ATP synthase is responsible for the ATP production and down-regulation of ATP5D is implied in the insufficient ATP production and cardiac hypertrophy.

Peroxisome proliferator-activated receptor-γ coactivator 1 α (PGC-1α) interacts with several members of the nuclear receptor superfamily, including ERR, NRF-1 and PPARα. PPARα is a cardiac-enriched member of the PPAR family known to control mitochondrial FAO enzyme expression[9,10]. Orphan nuclear receptors ERRa, is involved in substrate utilization, energy production and transportation across the mitochondrial membranes[11,12]. Therefore, PGC-1α is vital for the heart to meet increased demand for ATP and works well in response to physiological stimuli[13]. PGC-1α is considered responsible for the metabolic shift from fatty acid oxidation to glucose oxidation. This conclusion is in agreement with the observation that PGC-1α knockout mice experience a decrease in mitochondrial fatty acid oxidation and oxidative phosphorylation with preserved cardiac function[6]. In addition, PGC-1α has been shown to regulate the expression and activity of ATP5D, subsequently contributing to modulation of energy biosynthesis.

NF-κB activation plays key role in the development of myocardial hypertrophy[14,15]. NF-κB activation mediated translocation of p65, subunit of NF-κB into unclear fraction from cytoplasm regulates the targeted genes in the development of cardiac hypertrophy[14]. Recent data have demonstrated NF-κB activation resulted in reduced PGC-1α and Pyruvate dehydrogenase kinase 4 (PDK4) expression[16,17], contributing to cardiac metabolism in the process of cardiac hypertrophy[18,19]. Furthermore, inhibition of NF-κB also ameliorate cardiac fatty acid oxidation, achieving a better improvement in the prevention or inhibition of this pathological process[20].

Astragaloside IV(ASIV) is the major active ingredient extracted from the root of Astragalus membranaceus (Fisch) Bge, which has been widely used for cardiovascular diseases[21,22]. Several studies have demonstrated the potential protective effect of ASIV on energy metabolism[23]. We previously demonstrated cardiac protective role of ASIV in Iso-induced cardiac hypertrophy, which is at least partly attributed to the inhibition of TLR4/NF-κB signaling pathway[21]. The present study aimed to investigate the effects of ASIV on mitochondria derived energy biosynthesis and to test whether NF-κB/PGC-1α signaling pathway gets involved in the regulation of energy biosynthesis.

## Materials and Methods

### Materials

Astragaloside IV (purity >98%) was purchased from CHENGDU CONBON BIO-TECH CO.,LTD (Chengdu, China). Isoproterenol hydrochloride (MW 247.72, CAS N5984–95–2), Parthenolide (MW 248.32, CAS N20554–84–1) were purchased from Sigma (St, Louio, USA). The Enzyme-linked immunosorbent assay (ELISA) kits for free fatty acids (FFA), adenosine triphosphate (ATP), adenosine monophosphate (AMP) were from R&D Systems (Minneapolis, MN,USA). Antibodies against ATP5D, p65, PGC-1α, lamin B and β-actin were from abcam (Cambridge, MA, USA).

### Animal experiment

This study was carried out in strict accordance with the recommendations in the Guide for the Care and Use of Laboratory Animals of the National Institutes of Health. The protocol was approved by the Committee on the Ethics of Animal Experiments of the Liaoning Medical University (Permit Number: LMU-2013–368), China. Thirty six-week-old male Sprague-Dawley rats (180–200 g) were purchased from Animal Centre of the Liaoning Medical University (license number: SCXK 2009–0004). All rats were housed in a temperature-controlled room (25.0°C± 0.2°C) in a Specific Pathogen Free laboratory, with a 12-h/12-h light/dark photoperiod and 50% humidity. The rats were allowed free access to food and water. Rats were randomly divided into 3 groups (n = 10): (1) Control, rats received vehicle (1% Sodium Carboxymethyl Cellulose (CMC), 1ml, i.g.); (2) Iso, rats received isoproterenol injections (10mg×kg^−1^×day^−1^, i.p.); (3) Iso+ASIV, rats received isoproterenol injections and ASIV (80mg×kg^−1^×day^−1^, i.g). In order to investigate the preventive effects of ASIV, ASIV treatment started 2 weeks before Iso injection and lasted 4 weeks. ASIV were dissolved in 1% CMC solution. Control rats received the same volume of CMC (i.g.) and physiological saline (i.p.).

### Cell experiment

Neonatal rat ventricular myocytes (NRVMs) were isolated and cultured as described previously[24]. The experiments with NRVMs were performed after 24–48 h culture when synchronously contracting cells were observed. Briefly, the media was changed to serum-free Dulbecco’s modified Eagle’s medium (DMEM, Gibco Chemical Co) supplemented with 1% penicillin–streptomycin (Invitrogen) 24h before ASIV treatments. In this study, Iso (10μM) was used to stimulate myocardial hypertrophy. NRVMs were assigned to 4 groups: (1) Control group; (2) Iso (10μM) group; (3) Iso+ASIV (100μM) group and (4) Iso+parthenolide (10μM) group. Parthenolide (Par) is a specific NF-κB inhibitor. ASIV and Par were dissolved in dimethylsulfoxide (DMSO) and the final DMSO concentration did not exceed 0.1% (v/v). Before treatment with Iso, NRVM were incubated with ASIV and Par for 30 minutes. 48h later, cell surface area and ANP and BNP mRNA expressions were used as indices of hypertrophy.

### Small interference RNA

The small interference RNA (siRNA) sequences against rat p65 subunit were purchased from Santa Cruz Biotechnology (China). The scrambled sequence was used as a negative control (NC). Small interference RNA (100 nmol/L) was transiently transfected into NRVMs by adding it to lipofectamine 2000 (Invitrogen) according to the manufacturer’s instructions. The knockdown of p65 protein expression was confirmed by western blot analysis (data not shown). p65 siRNA or NC transfected NRVMs were assigned to 3 groups: (1) NC group; (2) Iso (10μM) +NC group; (3) Iso+p65 siRNA group. 48h later, surface area and protein expression of PGC-1α in cells were determined.

### Assessment of hemodynamics and heart weight index

All animals were anaesthetized with a 20% urethane (0.5ml/100g, i.p.) at the end of experiment. The right carotid were cannulated with a polyethylene catheter and then inserted into the left ventricle cavity, and the signals were recorded on an eight-channel direct-writing oscillograph (RM-6000, Nihon Kohden Kogyo Co., Ltd, Japan)[25]. The left ventricular systolic pressure(LVSP), left ventricular end-diastolic pressure(LVEDP), the maximal rate of left ventricular systolic and diastolic pressure (±dp/dtmax) were recorded using AcqKnowledge 3.5.7 software (BiopacSystems Inc., Santa Barbara, CA, USA). Then all animals were sacrificed by complete collection of the blood through right carotid cannula and the heart were immediately harvested, rinsed in ice-cold 0.9% NaCl solution, dissected and weighed. The heart-weight index (HW/BW), the left ventricle-weight index (LVW/BW) were calculated separately. The hearts were used for the following experiments.

### Histological analysis

Hearts were fixed in 4% formaldehyde and embedded in paraffin. The heart tissues were then sectioned (5μm) and stained with hematoxylin and eosin (HE). Myocyte surface area was measured and analyzed with LAS Software (V4.3) (Leica, Germany).

### Immunofluorescence and cell surface area determination

NRVMs were fixed with 4% paraformaldehyde and treated with 0.5μM rhodamine-labeled phalloidin as described previously[22]. Cell surface area was measured and analyzed with LAS Software (V4.3) (Leica, Germany).

### Cellular protein content

Total protein content of NRVMs was determined by Bradford assay against a BSA standard[26]. NRVMs with various treatments were cultured for 48h, washed with PBS and dissolved in 0.5ml 1% sodium dodecylsulphate (SDS) following the instruction of Bradford assay.

### ELISA assay

Heart issues and cells were used for measuring the levels of ATP, AMP and FFA by Enzyme-linked immunosorbent assay (ELISA) kits according to the manufacturer’s protocol[13].

### Real-time RT-PCR analysis

The mRNA expression levels were analyzed by quantitative real time RT-PCR using the BioRad iQ5 Real Time PCR system (BioRad Company). Total RNA from tissues or cells was extracted with TRIzol reagent (Invitrogen). The first strand cDNA was synthesized using AMV reverse transcriptase (TaKaRa, Dalian, China). Amplification was performed according to the manufacturer’s instructions using the SYBR Premix Ex Taq kit (TaKaRa, Dalian, China). The cDNA was denatured at 95°C for 5 seconds followed by 40 PCR cycles (95°C, 5 s; 60°C, 30 s). All results were repeated in four independent experiments. The relative level of mRNA was calculated by the comparative C_T_ method with GAPDH mRNA as the invariant control. The sequences of the primers (Invitrogen Biotechnology, Shanghai, China) used are: ANP: forward, 5’- ATG GGC TCC TTC TCC ATC AC-3’ and reverse, 5’- TTT CTC CTC CAA GGT GGT C-3’; BNP: forward, 5’-GGG CTG TAC CGG GCT GAG GTT-3’and reverse, 5’-AGT TTG TGG CTG GAA GAA TAA GA-3’ GAPDH: forward, 5’- CCA TCT TCC AGG AGC GAG ATC-3’ and reverse, 5’- GCC TTC TCC ATG GTG GTG AA-3’.

### Western blot

Using a commercial available Nuclear and Cytoplasm Extraction Kit (Active Motif), nuclear proteins were isolated from left ventricles of rats or NRVMs. The proteins (20 μg) were separated by SDS-PAGE on 10% separation gels, transferred to Hydrophobic Polyvinylidene (PVDF) membranes, and blocked with TBST (Tris-buffered saline with Tween-20) containing 5% non-fat dry milk. Primary antibodies of p65 (1:500 dilution), PGC-1α (1:1000 dilution), ATP5D (1:1000 dilution), β-actin (1:500 dilution) and lamin B (1:1000 dilution) were incubated at 4°C overnight. Then membranes were incubated with horseradish peroxidase-linked secondary antibodies (anti-rabbit, anti-mouse IgG). Detection was performed with chemiluminescence reagents (Amersham Biosciences). The results were quantified by the Quantity One software (Bio-Rad Laboratories, Hercules).

### Statistical analysis

Results are presented as mean ± SD for each group. Statistical analysis was performed by *one-way ANOVA* and the *Student-Newman-Keuls test* using SPSS 16.0 software; A *P*<0.05 was considered statistically significant.

## Results

### ASIV improves cardiac hemodynamics in rats

We previously reported that ASIV protected rat from Iso induced cardiac hypertrophy. To investigate the mechanism underlying the protection, we firstly assessed the cardiac hemodynamics of rats treated with Iso alone or in combination with ASIV. The results showed that rats treated with Iso alone demonstrated an increase in LVEDP and decreases in LVSP, +d_P_/d_t_max and-d_P_/d_t_max compared with normal control rats. However, combination with ASIV significantly decreased LVEDP and increased LVSP, +d_P_/d_t_max and-d_P_/d_t_max of rat compared with Iso alone (Table 1), suggesting that ASIV improves the cardiac hemodynamics induced by Iso.

**Table 1:** ASIV improves the cardiac hemodynamics in rats.

Group	LVEDP (mmHg)	LVSP (mmHg)	dP/dt (mmHg s^−1^)	−dP/dt (mmHg s^−1^)
Control	7.9±0.7	128.5±2.2	6157.4±201.2	4352.1±220
Iso	16.9±0.7**	115.8±2.5**	4340.5±213.8**	3081.9±282.5**
Iso+ASIV	10.2±0.6^##^	127.1±2.5^##^	5648.4±177.8^##^	4020.5±296.6^##^

The cardiac hemohynamics were measured as described in Materials and Methods section. Data are presented as mean ±S.D. (n = 8).

**P<0.01 *vs* control;

^##^ *P*<0.01 *vs* Iso.

LVEDP, Left ventricular end-diastolic pressure; LVSP, Left ventricular systolic pressure; +d_p_/d_t_, Maximal positive time derivative of developed pressure; −d_p_/d_t_, Maximal negative time derivative of developed pressure. Iso: Isoproterenol; ASIV: Astragaloside IV.

### ASIV down-regulates the mRNA expression of ANP and BNP in hypertrophic heart

**Fig 1:**
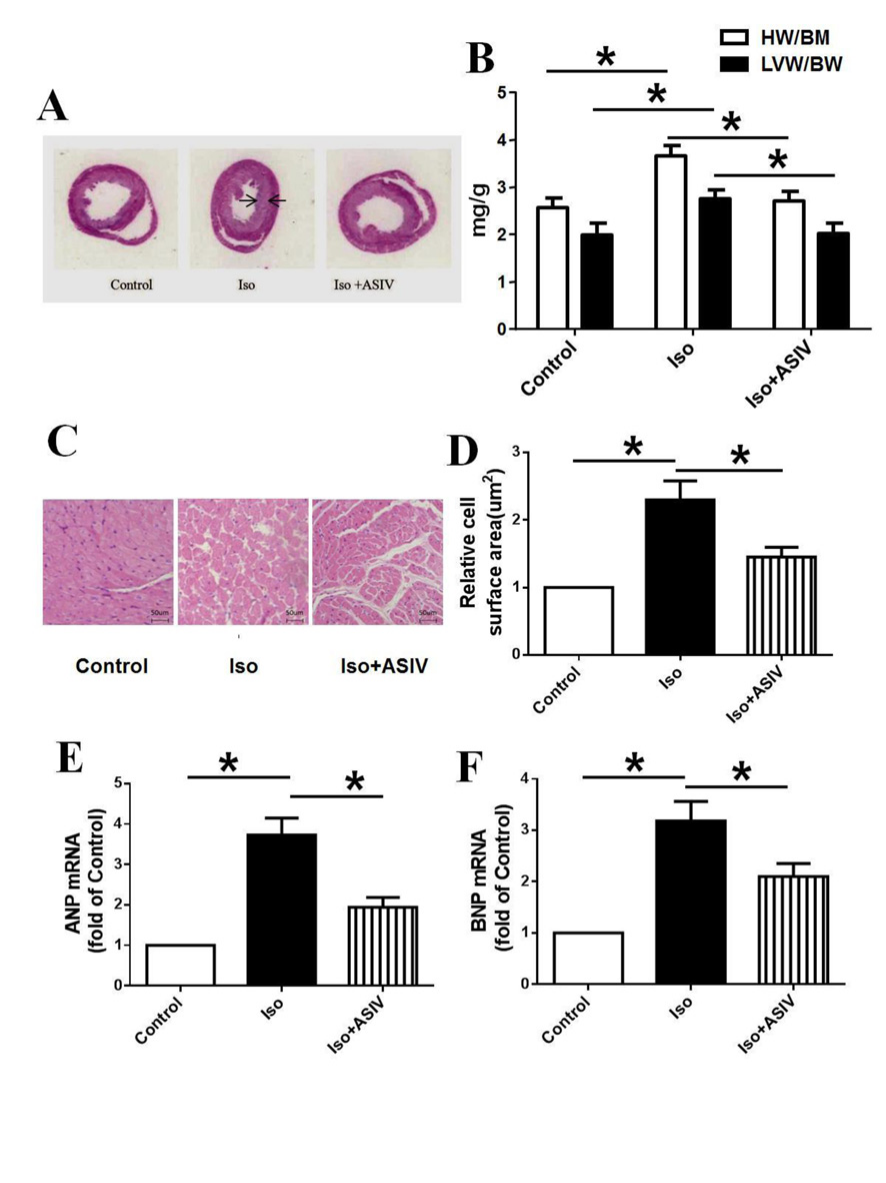
Effects of ASIV on index of cardiac hypertrophy and mRNA expression of ANP and BNP in hypertrophic heart. A: Left ventricular tissue section stained with H&E. B: ratios of heart weight/body weight (HW/BW) and left ventricular weight/body weight (LVW/BW). C: Representative of H&E staining of heart tissue; D: Statistical data of myocyte surface area shown in H&E staining measured and analyzed with LAS Software (V4.3) (Leica, Germany). E: mRNA expression of ANP. F: mRNA expression of BNP. Data are presented as mean ±S.D. n = 8 for A, B, C and D; n = 4 for E and F. *: *P*<0.05 is considered statistical significance.

ANP and BNP are two molecular markers of cardiac hypertrophy. We next investigated whether ASIV can regulate the mRNA expression of ANP and BNP in hypertrophic heart of rats. The results showed that treatment with Iso alone induced the cardiac hypertrophy represented by the thickness of the ventricular wall and the narrowness of the left ventricular cavity, increased the ratios of both HW/BW and LVW/BW and up-regulated the mRNA expression of ANP and BNP compared with normal control rats. However, combination with ASIV significantly reduced the thickness of the ventricular wall, attenuated the narrowness of the ventricular cavity, decreased the ratios of HW/BW and LVW/BW and down-regulated the mRNA expression of ANP and BNP compared with Iso alone (Fig. 1), suggesting that down-regulation of ANP and BNP at least partly explains the protection of ASIV on cardiac hypertrophy.

### ASIV protects NRVM from hypertrophy

**Fig 2:**
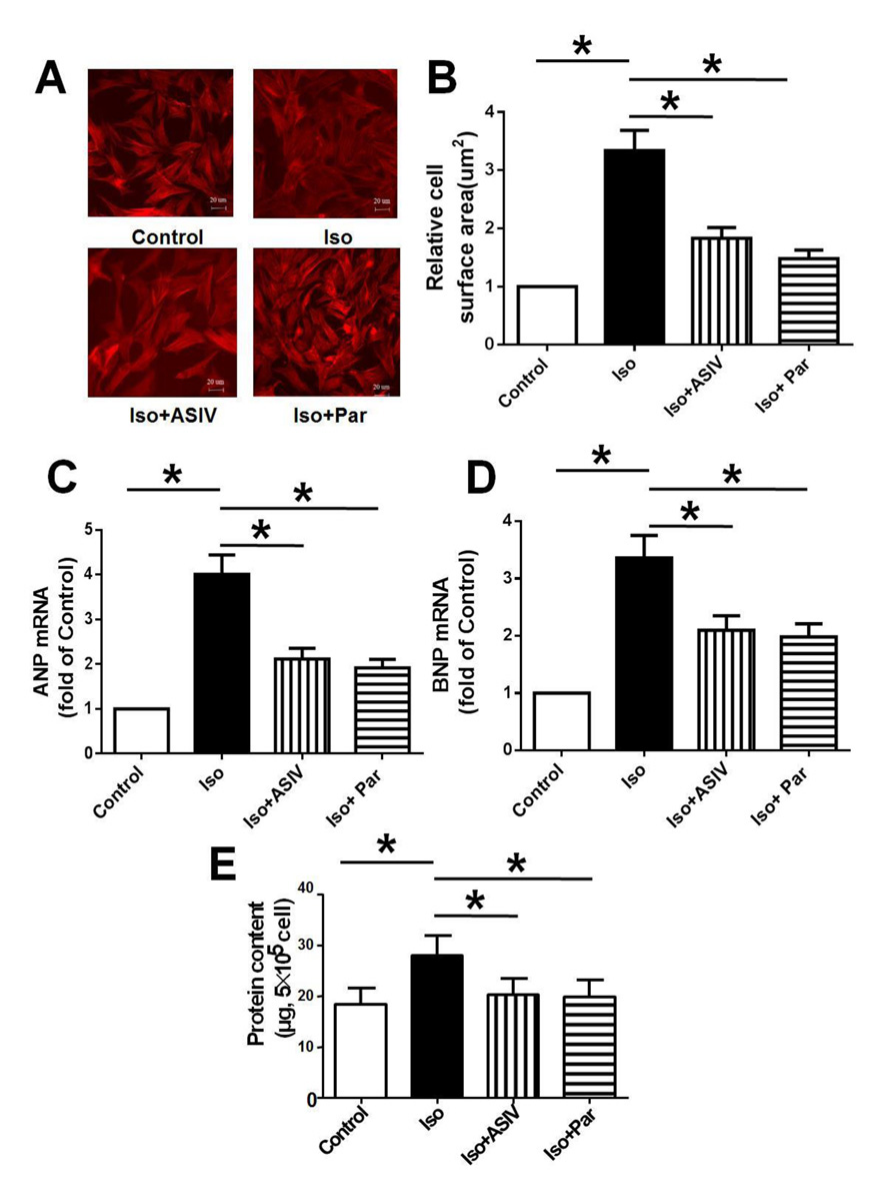
Effect of ASIV on Iso-induced NRVM cells. A: The cell morphology was visualized by immunofluorescence analysis. B: Cell surfaced area was measured and analyzed with LAS Software (V4.3) (Leica, Germany). C: mRNA expression of ANP. D: mRNA expression of BNP. E: Protein content. Data are presented as mean ±S.D. n = 4. *: *P*<0.05 is considered statistical significance.

To verify the protective effects of ASIV on cardiac hypertrophy *in vivo*, we cultured the NRVM cells and study the effects of ASIV on Iso induced hypertrophy *in vitro*. We found that NRVM treated with Iso alone demonstrated the increases in cell surface area, protein content and mRNA expression of ANP and BNP compared with normal control cells. Combination with ASIV resulted in significant decreases in cell surface area, protein content as well as mRNA expression of ANP and BNP compared with Iso alone (Fig. 2).

### ASIV corrects the dysfunction of cardiac energy biosynthesis

**Fig 3:**
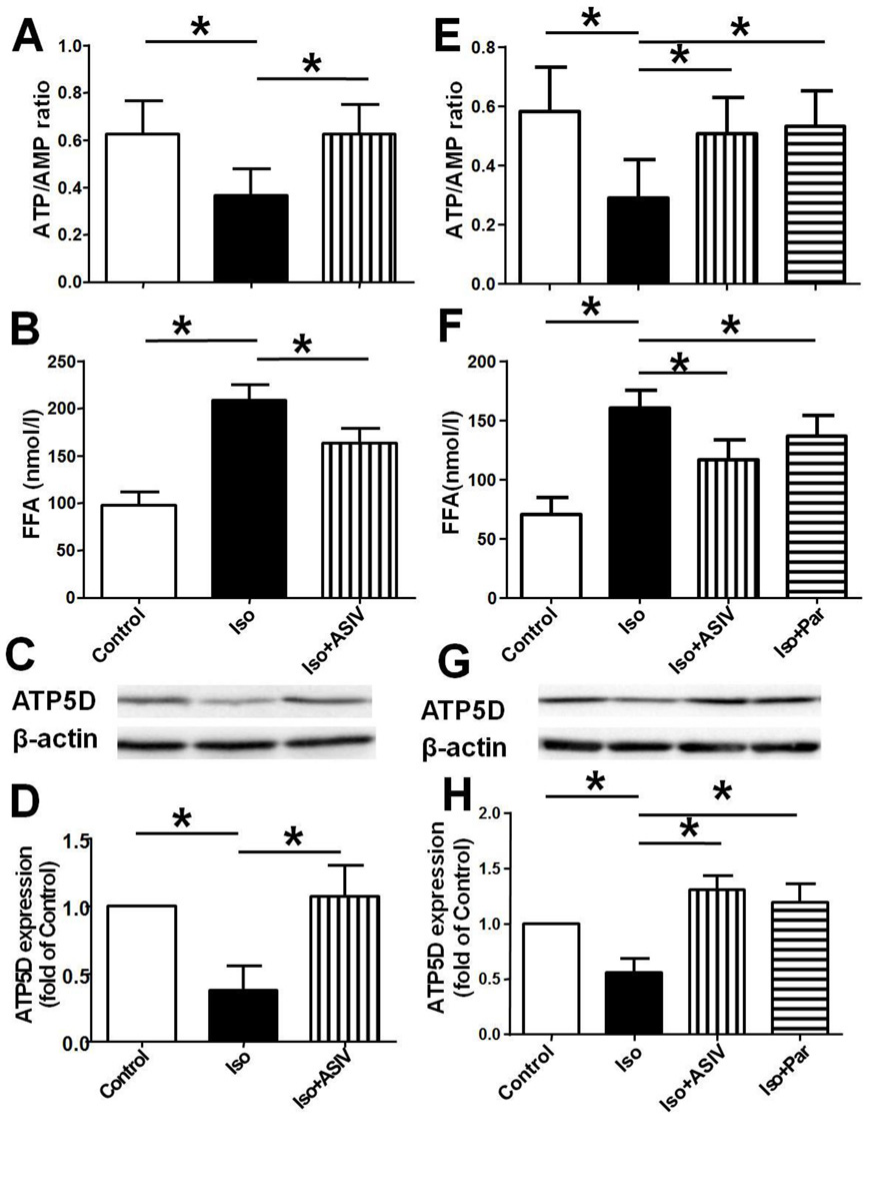
Effects of ASIV on Iso-induced changes in the energy biosynthesis and expression of ATP5D. A and E: ATP/AMP ratio in vivo or in vitro. B and F: FFA level in vivo or in vitro. C and G: Representative of western blot photograph of ATP5D in vivo or in vitro. D and H: Statistical data of protein expression of ATP5D. Data are presented as mean ±S.D. n = 8 for A, B, C and D; n = 4 for E, F, G and H. *: *P*<0.05 is considered statistical significance.

Dysfunction of energy biosynthesis in cardiac mitochondria contributes to the hypertrophy. ATP5D, subunit of ATP synthase, is one of the enzymes responsible for the ATP synthesis in mitochondria. To explore the mechanisms underlying the protection of ASIV on cardiac hypertrophy, we examined the effects of ASIV on Iso induced dysfunction of cardiac energy biosynthesis of rats and NRVM. The results showed that treatment with Iso alone decreased the ratio of ATP/AMP and increased FFA content in both heart tissue and NRVM compared with normal control rats or cells, meanwhile decreased the protein expression of ATP5D in NRVM compared with control NRVM. However, treatment with ASIV significantly increased the ratio of ATP/AMP and reduced the FFP content in both rats and NRVM, and increased the protein expression of ATP5D in NRVM compared with Iso alone (Fig. 3). The results suggest that ASIV prevent cardiac hypertrophy at least partly through improvement of cardiac energy biosynthesis which might be attributed to the up-regulation of ATP5D protein expression.

### Effect of ASIV and parthenolide on expressions of NF-κB and PGC-1α

**Fig 4:**
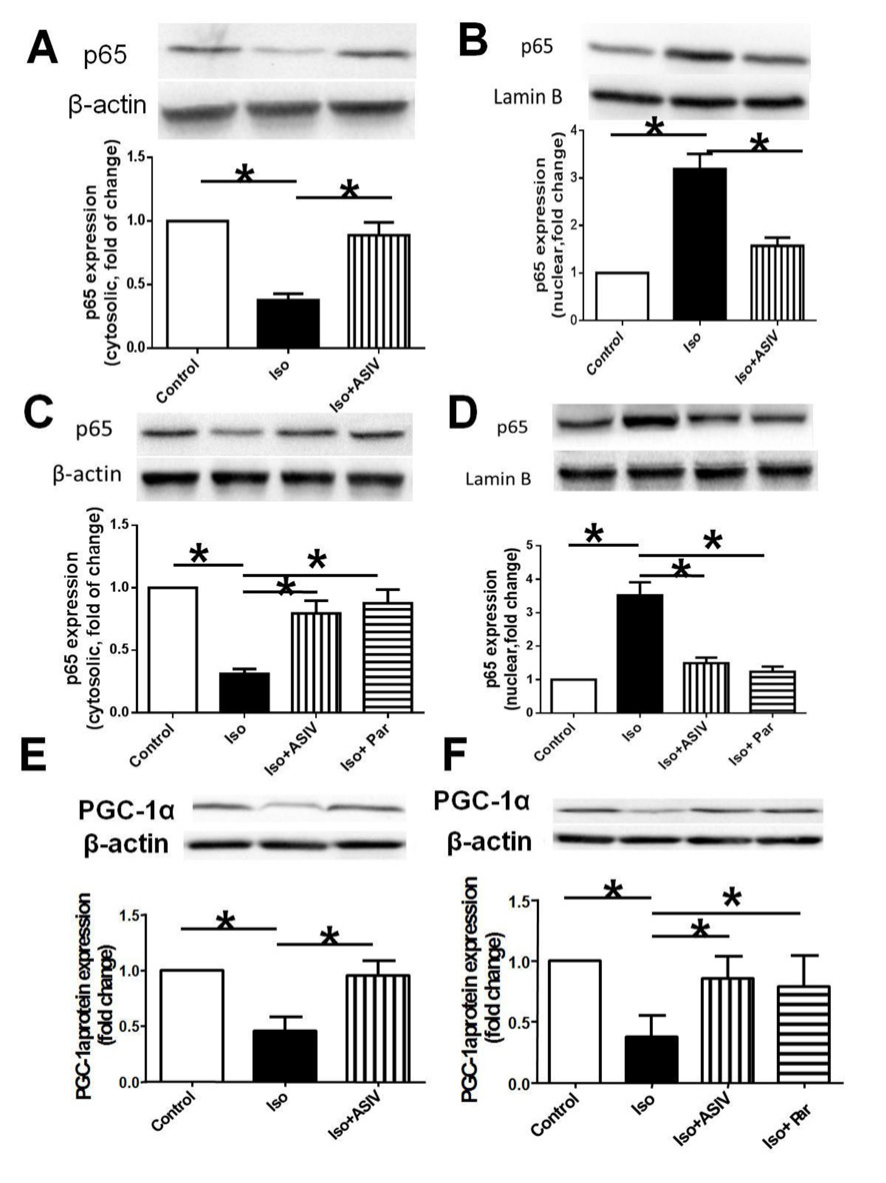
Effects of ASIV on p65 and PGC-1α protein expression measured by Western blot. A and B: p65 expression in cytoplasm and nuclear fraction of heart tissue. C and D: p65 expression in cytoplasm and nuclear fraction of NRVMs.B: E and F: PGC-1α expression in heart tissue and NRVMs. Data are presented as mean ±S.D. n = 4. *: *P*<0.05 is considered statistical significance.

NF-κB/PGC-1α signaling is closely related to the regulation of ATP5D, subsequently affecting the energy biosynthesis. We finally examined whether NF-κB/PGC-1α signaling gets involved in the correction of cardiac energy biosynthesis dysfunction by ASIV, subsequently contributing to the protection against cardiac hypertrophy. The results showed that treatment with Iso alone decreased the protein expression of p65, the subunit of NF-κB in cytoplasm while increased the expression of p65 in nuclear fraction, and decreased PGC-1α protein expression in both rat and NRVM compared with normal control rat or cells. However, combination of ASIV significantly decreased the p65 expression in nuclear fraction while increased the expression of p65 in cytoplasm meanwhile increased the PGC-1α expression compared with Iso alone (Fig. 4). Parthenolide, the specific inhibitor of p65, exerted similar effects as ASIV on PGC-1α expression in cells. The results suggested that ASIV exhibits the improvement of dysfunction in cardiac energy biosynthesis at least partly through regulation of NF-κB/PGC-1α signaling.

### Knockdown of p65 using siRNA decreased surface area and PGC-1α protein expression of NRVMs

**Fig 5:**
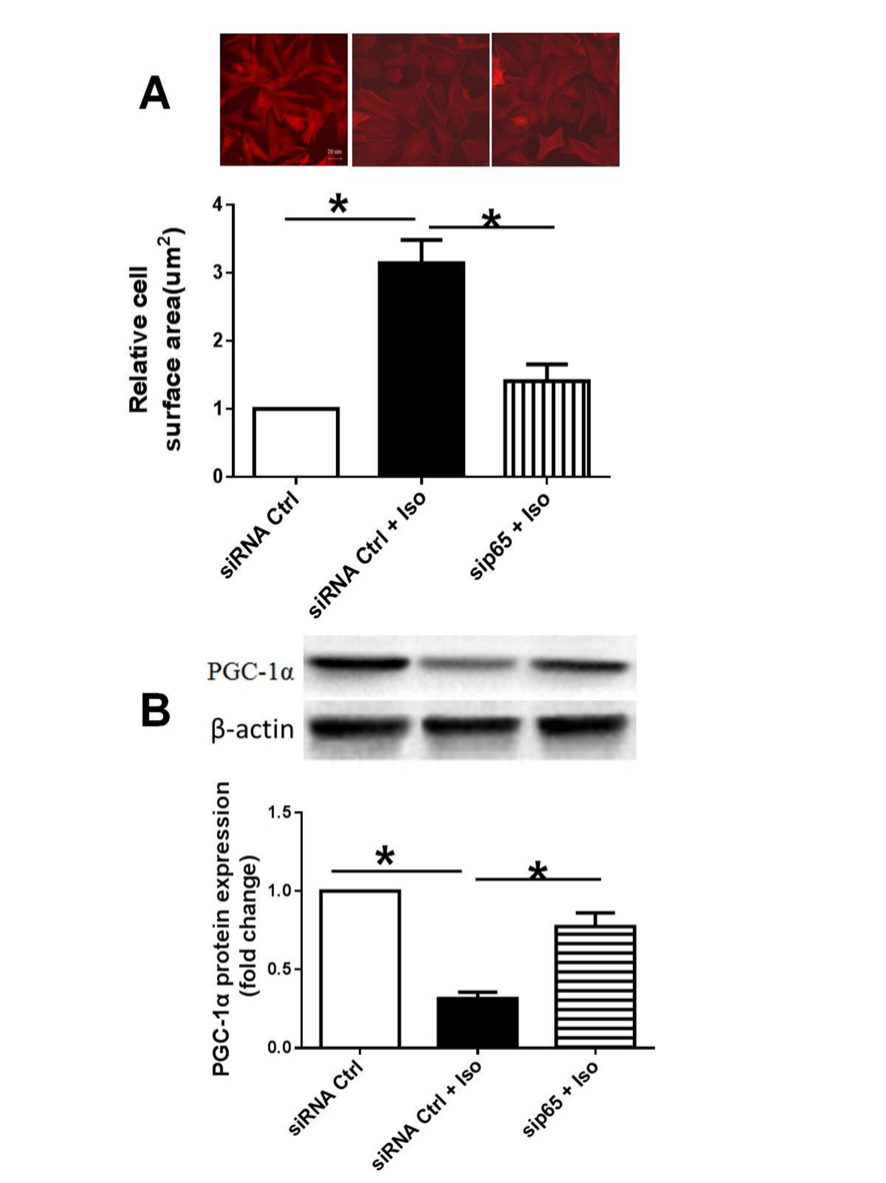
Effect of p65 knockdown on surface area and PGC-1α protein expression. A: representative cell morphology (upper panel); Statistical data of cell surface area (lower panel). B: PGC-1α protein expression in NRVMs.

To confirm the down-regulation of PGC-1α by NF-κB activation, we knocked down the p65 using siRNA to observe the effect of inactivation of NF-κB on PGC-1α protein expression in NRVMs treated by Iso. We found that knockdown of p65 decreased the surface area and increased the protein expression of PGC-1α in NRVMs compared with the Iso alone (Fig. 5), suggesting the importance of NF-κB activation in the myocytes hypertrophy and down-regulation of PGC-1α.

## Discussion

We reported in the present study that ASIV improves the cardiac hemodynamics, down-regulates mRNA expression of ANP and BNP, corrects the dysfunction of energy biosynthesis, up-regulates the protein expression of ATP5D and PGC-1α, and inhibited the translocation of p65 into nuclear fraction from cytoplasm in rats and NRVMs treated with Iso. The findings confirm and expand the results reported previously[21], in which we found that ASIV protects against Iso-induced cardiac hypertrophy in terms of ratios of HW/BW and LHW/BW as well as ANP and BNP mRNA expression. In the present study, we additionally examined the cardiac hemodynamics and verified the protective role of ASIV in cardiac hypertrophy based on the improvement of hemodynamics.

Cardiac hypertrophy is commonly viewed as a compensatory response, accompanied by neurohormonal activation, inflammatory responses and metabolic remodelling[1,2]. The energy metabolic reprogramming are characterized with mitochondrial dysfunction, shift of cardiac substrate utilization, and alterations in high-energy phosphate metabolism[17]. Clinical research revealed that ASIV had a variety of therapeutic effects on failure heart such as metabolism modulation, anti-inflammation, anti-oxidation, anti-thrombosis. The metabolism modulation of ASIV involved energy changes and ATPase activity[23,27]. It is known that ATP is required for myocardial pump function. The energy demands for the heart are immense. Normally, myocardial ATP is mainly generated from mitochondrial oxidation of fatty acids, which accounts for 60–90% of the total energy production[28]. Numerous experimental and clinical studies indicates that the hypertrophied heart is characterized by a shift in the utilization of substrate, from fatty acids oxidation towards glycolysis[29,30]. As the disease stage progressed, the presumably adaptive increase in glycolysis is not sufficient to meet ATP demand, following marked intracellular lipid accumulation and leading to lipotoxic cardiomyopathy[18]. In the present study, we found ASIV reduced the accumulation of FFA and increased the ratio of ATP/AMP, implying ATP synthesis by the mitochondria was increased. Consistent with this result, the expression of ATP5D, which is a ATP synthase subunit and contributes to the synthesis of ATP, was up-regulated by ASIV, suggesting the beneficial role of ASIV in Iso-induced cardiac metabolic reprogramming.

The directive molecules mechanism of compromised energy metabolism were associated with PGC-1α or PPARs[9]. It has been reported that PGC-1α coactivates members of the PPAR nuclear receptor transcription factor family, and the regulation of cardiac PPAR/PGC-1 complex is important for ATP production, so as to maintain normal cardiac function[31]. Therefore, PGC-1α plays a crucial role in the hypertrophic response. Furthermore, the recent research show that the increased physical interaction between NF-κB p65 and PGC-1α leads to a metabolic disturbances in cardiac cells. Our previous study has been demonstrated that ASIV could significantly decrease the NF-κB protein expression[21]. Therefore, ASIV was hypothesized to increase PGC-1α based on the inactivation of NF-κB, consequently to relieve the metabolic disorder in Iso-induced myocardial hypertrophy.

To explore the mechanism underlying the metabolism modulation action of ASIV, we examined the levels of NF-κB and PGC-1α proteins. It has been shown that PGC-1α activates PPARα to induce FAO pathway enzymes[32]. Abnormal expression of NF-κB and PGC-1α are associated with myocardial metabolism derangements[32,33]. The results in the present study showed that ASIV inhibited translocation of p65, subunit of NF-κB into nuclear fraction from cytoplasm and increased PGC-1α protein expression in heart tissues or NRVMs compared with Iso alone, demonstrating that the modulation of energy by ASIV was accompanied by the regulation of NF-κB and PGC-1α expression.

Amounting evidence showed that inhibition of NF-κB may be sufficient to prevent cardiac hypertrophy[32] and PGC-1α activates expression of genes involved in FA uptake and oxidation when overexpressed in cardiac myocytes. In H9c2 myocardiac cells, TNF-α activates the expression of PGC-1α through NF-κB, resulting in metabolic disorders[25]. And the binding of NF-κB p65 and PGC-1α is demonstrated both in vitro and vivo, leading to metabolic shift and metabolic disturbances[34,35]. The data suggest that NF-κB/PGC-1α complex axis may contribute to the metabolism reprogramming in heart hypertrophy. In the present study, the downregulation of PGC-1α expression, which is a major regulator of key enzymes needed for high-efficiency ATP production, were partially restored by ASIV. And the results in vitro revealed that incubated with ASIV or NF-κB inhibitor, parthenolide, the cells showed no significant difference in PGC-1α mRNA level. The potential pharmacological action of ASIV to ameliorate energy metabolism disorder may be related to NF-κB/PGC-1α pathway.

In summary, our results demonstrate for the first time that ASIV could prevent Iso-induced hypertrophy and alleviate energy metabolism disorder, partly through NF-κB/PGC-1α pathway. These findings strengthen the therapeutic rationale for ASIV in the heart disease.
